# How can healthcare organizations implement patient-centered care? Examining a large-scale cultural transformation

**DOI:** 10.1186/s12913-018-2949-5

**Published:** 2018-03-07

**Authors:** Barbara G. Bokhour, Gemmae M. Fix, Nora M. Mueller, Anna M. Barker, Sherri L. Lavela, Jennifer N. Hill, Jeffrey L. Solomon, Carol VanDeusen Lukas

**Affiliations:** 1 Center for Healthcare Organization and Implementation Research, ENRM Veterans Affairs Medical Center, Bedford, MA USA; 2 Department of Health Law, Policy & Management, Boston University School of Public Health, Boston, MA USA; 3 Department of Behavioral and Community Health, University of Maryland School of Public Health, College Park, MD USA; 4 Center for Innovation for Complex Chronic Healthcare (CINNCH), Department of Veterans Affairs, Edward Hines Jr. VA Hospital, Hines, IL USA; 5 Department of Physical Medicine and Rehabilitation, Northwestern University Feinberg School of Medicine, Chicago, IL USA; 6 Independent Research Consultant (formerly VA), Chicago, IL USA

**Keywords:** Patient-centered care, Organizational change, Qualitative methods, Leadership

## Abstract

**Background:**

Healthcare organizations increasingly are focused on providing care which is patient-centered rather than disease-focused. Yet little is known about how best to transform the culture of care in these organizations. We sought to understand key organizational factors for implementing patient-centered care cultural transformation through an examination of efforts in the US Department of Veterans Affairs.

**Methods:**

We conducted multi-day site visits at four US Department of Veterans Affairs medical centers designated as leaders in providing patient-centered care. We conducted qualitative semi-structured interviews with 108 employees (22 senior leaders, 42 middle managers, 37 front-line providers and 7 staff). Transcripts of audio recordings were analyzed using a priori codes based on the Consolidated Framework for Implementation Research. We used constant comparison analysis to synthesize codes into meaningful domains.

**Results:**

Sites described actions taken to foster patient-centered care in seven domains: 1) leadership; 2) patient and family engagement; 3) staff engagement; 4) focus on innovations; 5) alignment of staff roles and priorities; 6) organizational structures and processes; 7) environment of care. Within each domain, we identified multi-faceted strategies for implementing change. These included efforts by all levels of organizational leaders who modeled patient-centered care in their interactions and fostered willingness to try novel approaches to care amongst staff. Alignment and integration of patient centered care within the organization, particularly surrounding roles, priorities and bureaucratic rules, remained major challenges.

**Conclusions:**

Transforming healthcare systems to focus on patient-centered care and better serve the “whole” patient is a complex endeavor. Efforts to transform healthcare culture require robust, multi-pronged efforts at all levels of the organization; leadership is only the beginning. Challenges remain for incorporating patient-centered approaches in the context of competing priorities and regulations. Through actions within each of the domains, organizations may begin to truly transform to patient-driven care.

## Background

Since the Institute of Medicine identified patient-centered care (PCC) as a critical aspect of quality, there have been many initiatives to promote PCC in healthcare organizations [1]. PCC represents a shift from traditional, paternalistic, provider-driven, disease-focused approaches towards healthcare systems that ensure patients—including their preferences, needs, desires and experiences—are fully integrated into every phase of medical consultation, treatment and follow-up [2]. PCC includes empowering patients, focusing on the patient-provider relationship, and enabling providers to partner with patients to better meet patient goals. Research has demonstrated the effectiveness of PCC innovations in improving patient experiences, trust, care quality, chronic disease management and outcomes [2–6]. Yet, despite many years of discussion about PCC and its impact on health, healthcare remains largely provider-focused and disease-driven.

Little is known about how best to transform a traditionally provider-centric care system into one where patient preferences and goals drive care and sustain this change [7–9]. One study of hospitals identified several key organizational and patient-level practices associated with high scores on patient experiences on inpatient units, including a focus on nursing practices, leadership rounds and accountability for clinician behavior [10]. Other studies looking at facilities excelling in patient experience identified key practices for fostering PCC, including strong leadership commitment, communicating a strategic vision, systematic measurement and feedback and having accountability and incentives for providing PCC [11, 12]. Yet our understanding of organizational practices necessary to shift the culture of a large healthcare organization to implement and embrace PCC remains incomplete.

Implementation science provides conceptual frameworks for understanding how healthcare organizations can implement evidence-based practices. Frameworks such as the Consolidated Framework for Implementation Research (CFIR) typically focus on discrete, evidence-based clinical practices, yet are applicable to assessing implementation of broader system-level PCC transformation [13]. The CFIR identifies a menu of constructs within five areas (intervention characteristics, inner setting, outer setting, individual characteristics, and process) that interact to influence implementation. Examples include: strength of evidence for an innovation (knowledge and belief about the innovation); innovation fit within the organization’s practices (adaptability, trialability, relative priority); organizational readiness to implement change (implementation climate, relative priority); roles of leadership (engagement and resource allocation); and engagement of staff. Examining these constructs may provide a foundation for understanding how organizations foster broad transformational efforts of a healthcare system, such as shifting towards a culture of PCC.

In 2010, the Department of Veterans Affairs (VA) embarked on a mission to transform VA healthcare to provide personalized, proactive, patient-driven healthcare [14]. Senior VA leadership recognized that change for such a large complex organization would be a lengthy process requiring on-going piloting and testing innovations, evaluating outcomes, and deploying effective strategies across the system. Office of Patient-Centered Care and Cultural Transformation (OPCC&CT) provided funds to designated VA medical centers considered early leaders in PCC. These facilities became “Centers of Innovation” (COIs), living laboratories of PCC innovations spanning the spectrum from environmental changes, to personalized health planning, to integrative medicine. One front line provider at a COI defined patient-centered care:

“*[It] is about empowering patients to pursue wellness in service of their life goals and their own values. It’s about strengthening the patient-provider relationship. It’s about empowering providers in the work setting so that they have the best tools available … so that they are able to partner with patients”.*

Yet how to get this idealization of PCC into practice remains uncertain. This paper describes a qualitative study of four early COIs to inform VA leadership about how best to catalyze and sustain change across the system. Using the CFIR as a guide, we evaluated COI activities to identify key organizational factors that fostered or impeded the implementation of PCC.

## Methods

### Study design

We conducted qualitative site visits in 2013 at four large VA medical centers in different regions of the country which were established COIs. All four were urban medical centers, considered to be high complexity - that is they provide a wide range of services (i.e. surgical, inpatient care, outpatient care, mental health and substance abuse services, residential and extended care, have educational and research missions and serve complex Veteran populations). These medical centers had been selected to be COIs based on a past track record of engaging with OPCC&CT and implementing some level of PCC innovation in their facilities. All centers had received prior pilot funding from OPCC&CT for targeted initiatives. They had engaged with an outside consultant organization who is a leader in transforming the culture of healthcare organizations to patient-centeredness. The centers were at various stages, with 2 centers further along and having embraced PCC for over 8 years, while 2 centers had more recent changes in leadership which chose to focus on PCC as a critical initiative within the prior 3 years. The institutional review board at the Bedford Veterans Affairs Medical center designated the study as quality improvement and was IRB-exempt, thereby waiving the need for review or participant consent for the study. The directors of the medical centers involved in the study provided permission for the evaluation team to conduct the study.

### Participants

In accordance with qualitative notions of validity [15] we sought to gain the perspectives of a range of key participants with a role in PCC innovation at the COIs. We worked closely with medical center leadership, including the medical center director or associate director and PCC coordinators, to identify individuals they considered critical to the implementation of PCC innovation. Their participation in the process reflected the investment of the facility in PCC. They identified key informant providers, administrators and front line staff for interviews who played a critical role in the planning and/or roll-out of PCC innovations and cultural transformation efforts. Potential participants were contacted via e-mail explaining the purpose of the site visit and inviting participation.

### Data collection procedures

Teams of two researchers conducted 3–4 day site visits from January–April 2013. We conducted semi-structured key informant interviews. Although most interviews were conducted individually, group interviews were conducted when the key informant brought others he/she felt were important to answering questions outlined in the e-mail invitation. For example, at one site the integrative medicine head invited other staff involved in integrative medicine programs.

Interview protocols – one for leadership and one for front-line staff – were informed by CFIR constructs (Table 1). Topics included perceptions about PCC, roles of leadership, engagement of providers and patients, and challenges and facilitators to PCC innovation implementation. Interviews lasted approximately 1 hour. We audio-recorded interviews with participants’ consent; when participants declined audio-recording, we took detailed notes.

**Table 1:** Interview Questions with Associated CFIR Constructs

Leadership Interview	CFIR constructs
I. Individuals’ background	Characteristics of Individuals
II. Perspectives on Patient-centered care. What is it?	Characteristics of Individuals
III. Pre-implementation experiences – how the organization came to focus on patient-centered care transformation and innovation. Impetus to change; challenges faced.	Inner Setting Intervention Characteristics Outer setting
IV. Evaluation of the initiative implementation – goals and how it differs from prior practice.	Intervention Characteristics
V. Description of implementation a. Training, engaging staff members, challenges and evaluation	Intervention Characteristics Process
VI. Future of the program a. How will PCC innovation look in the future; what will make it work? b. How innovation meets goals of patients and staff. c. Assessment of success and of difficulty in transforming and why. d. What are your lessons learned?	Intervention Characteristics Process Outer setting
Staff Interview	CFIR constructs
I. Background of the individual	Characteristics of Individuals
II. Perspectives on patient-centered care. What is it?	Characteristics of Individuals
III. Pre-Implementation experiences Learning about PCC innovation, challenges faced; how the organization came to focus on patient-centered care transformation and innovation.	Inner Setting Intervention Characteristics
IV. Evaluation of the initiative implementation a. What is your understanding of the goals of this initiative? b. How is this different from what you were doing previously?	Inner Setting
V. Description of implementation Training received; fit of the initiative with clinical practice; how staff get engaged; patient perceptions of the innovation; feedback received.	Intervention Characteristics Process
VI. Future of the program a. How will PCC innovation look in the future; what will make it work. b. How innovation meets goals of patients and staff. c. Assessment of success and of difficulty in transforming and why. d. What are your lessons learned?	Intervention Characteristics Process Inner setting

### Data analysis

Audio-recordings were reviewed and transcribed. Team members reviewed 5 transcriptions jointly (2–4 investigators), and coded them using the established CFIR codebook [13]. Discussions regarding applicability of specific codes resulted in agreement on specified coding definitions. Additional concepts arose through inductive analysis and led to generation of 10 additional codes to capture areas important to culture change [16]. Evaluation team members worked iteratively to ensure findings accurately reflected data to reach consensus on a comprehensive shared codebook.

Constructs of the CFIR that emerged as predominant throughout the sites were identified. “Memos” were created to document ongoing analysis and constant comparison processes [17]. To make our work salient to the VA policy makers in OPCC&CT, these were then synthesized along with emergent codes, into key domains impacting PCC and cultural transformation. Supporting subthemes were identified in each major domain.

## Results

A total of 108 participants from senior management (SM), middle management (MM), front-line providers (FLP) and other staff (OS) participated in individual or group interviews (Table 2).

**Table 2:** List of Participants*

ROLE	N
Senior Medical Center Leadership	
Medical Center Director Assistant Director Senior Management Team Chief of Staff Associate/Deputy Chief of Staff Chief Financial Officer Nursing Leadership	13 interviews; 22 people
Middle Management	
Patient-centered care Coordinators and Leaders Patient-centered care Committee members Service Chiefs Voluntary Service Information Technology Pharmacy Engineering Environmental Management Services Specialty clinical Service Anesthesiology Home based primary care Integrative and alternative medicine managers Educational Leaders Health Promotion and Disease Prevention Coordinator	23 interviews; 41 people
Frontline Providers	
Psychologists Physicians Recreational Therapists Nurses Dietitians Social Workers	34 interviews; 37 people
Other Staff	
Systems Redesign Coordinators Architects Interior Designers Public Affairs Officers Information Technologist Program Specialist	7 interviews; 7 people
TOTAL	*N* = 77 interviews or group interviews**;** 107 people

*All sites had participants who represented each of the following categories. In the ‘other’ category, some sites identified these individuals as important, while others focused more on the leadership, middle managers and front line providers

We found elements of all five main CFIR constructs were relevant for transforming care. These constructs, along with emergent codes, informed identification of seven domains that impacted PCC implementation: 1) leadership, 2) patient and family engagement, 3) staff engagement, 4) focus on PCC innovations, 5) alignment of staff roles and priorities, 6) organizational structures and processes, and 7) environment of care (Table 3).

**Table 3:** Seven Domains for Implementing Patient-Centered Care and Associated CFIR Constructs

Domain	CFIR broad construct	CFIR subconstruct
Leadership	Inner Setting	Leadership engagement Opinion Leaders Champions
	Process	Engaging
Patient and family engagement	Intervention Characteristics	Intervention Source Design quality and packaging
	Outer Setting	Patient needs and resources
Staff engagement	Intervention characteristics	Design quality and packaging
	Inner setting	Networks and communications Culture Implementation climate Relative priority Learning climate Access to knowledge and information
	Process	Engaging Opinion leaders Reflecting and evaluating
Focus on PCC innovations	Intervention characteristics	Intervention source Relative advantage
	Inner setting	Implementation climate Relative priority Learning climate Readiness for implementation Leadership engagement
	Characteristics of individuals	Self-efficacy
	Process	Opinion leaders Champions
Alignment of staff roles and priorities	Intervention characteristics	Adaptability Complexity
	Characteristics of individuals	Identification with organization Knowledge and beliefs about intervention Self-efficacy
	Inner setting	Networks and communication Culture and climate
Organizational structures and processes	Inner setting	Networks and communication Culture and climate
	Outer Setting	External policy & incentives
Environment of care	Inner setting	Culture and climate

### 1) Leadership

Leadership commitment to creating a PCC organization was critical to transforming care. This domain was mentioned by almost all participants, equally by leaders themselves and front-line staff. The importance of leadership was infused throughout the interviews, and marked as critical to move forward any initiatives. Two sites discussed that prior leadership had been less supportive and it was with a shift in leadership that PCC could move forward. It was important to have leaders who served as models for PCC and actively engaged staff in local PCC initiatives. Staff reported that PCC had to be a “*strategic priority, emphasized by the director”* through implicit endorsement of PCC and providing resources for initiatives. We identified several important themes within this domain.

#### Leaders must express support for PCC openly, consistently and frequently

Opinion leaders, clinicians, and other staff noted that consistent and frequent expressions of support for individual initiatives and the cultural transformation as a whole were integral to successful change.

*“I’d have to say the office of the director, without support from the top, you can forget about it, but our hospital director is awesome … mentions it at every single chance.” (MM).*

*“Having the full support from the leadership at this hospital, it’s been frankly shocking. I’m a frontline worker bee and to have our hospital director saying ‘this is what we want to do, this if the future, we have to transform our healthcare culture,’ sure makes a big difference.” (FLP).*

As seen here, leadership’s dedication to transformation was reinforced by talking with staff, motivating them to become equally dedicated.

Having senior leaders participate in each PCC subcommittee represented this ongoing support:

*“You’ve got to have leadership at the table … [making] decisions for it to move forward.” (SM).*

In this way, ideas for new initiatives and challenges could quickly be shared with senior decision makers for consideration.

#### Encouraging staff risk-taking and getting staff feedback fosters staff engagement

Leaders who encouraged risk, were open to new ideas, and actively sought feedback from staff facilitated staff buy-in and spread of PCC. One hospital director was described as “thinking outside the box,” encouraging individuals to “pilot” ideas and share lessons learned about what worked and did not work.

One participant noted:

*“I am supported and encouraged to be a risk-taker. If [I] look at things from a Veteran-centered perspective, I will be supported.” (MM).*

Another way leaders garnered input was by providing an “idea box” for staff to submit ideas for PCC activities. Staff were given feedback regardless of the feasibility of the innovation.

#### Leadership should model PCC for the staff and engage staff

Leadership was perceived as most effective when they modeled PCC in every interaction with staff and patients. Leaders set the expectation that staff have an emotional commitment to caring for Veterans, setting the stage for practice changes. One senior manager, describing the cultural shift for all employees said:

*“My role within VA …is certainly to model that behavior and to be sure …that we’re driving the initiative forward, …always focusing on the Veteran, and involving the Veteran in the decision making process.” (SM).*

One provider noted the importance of modeling at all leadership levels:

*“The executive leaders, the formal and informal leaders, the supervisors start off sort of being this shift, being this change and modeling and very intentionally choosing, behaving different in meetings and behaving different in every interaction. Because that’s how this grows and that’s how we teach everyone all the time, every day.” (FLP).*

Modeling PCC also came in the form of demonstrating staff-centeredness, such as respecting and engaging staff as individuals rather than just employees. One senior manager stated he actively listened to staff’s personal stories, concerns, and needs; doing so was believed to foster staff’s similar treatment of patients.

#### A Core cadre of leaders is needed to move PCC forward

Effective leadership meant the establishment of several core leaders actively involved in PCC, including a PCC committee with members having dedicated time to pursue PCC initiatives. Moreover, middle managers played important roles. One participant described how the chief of her service leads the team towards providing PCC:

*“She has this vision which includes relationships, staff engagement and empowerment. That’s just what she’s like…She just puts that into reality.” (FLP).*

#### Experiencing PCC fosters leadership engagement

Leadership became engaged in PCC transformation in two ways. First, several senior managers stated they visited state-of-the-art patient-centered hospitals; seeing the quality and environment of care at these facilities convinced them to initiate transformation in their own facilities.

Second, emotionally salient experiences made some leaders rethink the quality of care. One member of senior management described a clinical scenario in which he realized that *“providing good evidence-based care might not be sufficient.”* Reviewing a case where a Veteran had committed suicide, he reflected:

*“His diabetes was under control, his cholesterol was good, his CHF, all his numbers were good. We’re providing this man very, very good clinical care… There was something going on with this man that for whatever reason, through nobody’s fault we didn’t discover… It was one of those ‘AHA’ moments, you know?” (SM).*

For this leader, the nature of quality care had shifted from traditional clinical care performance measures to one in which attention to the patient as an individual was critical.

Leadership at several facilities described spending time listening to and learning from Veterans’ stories. One director discussed how hearing a Veteran’s story of combatting alcohol abuse with yoga opened his eyes to the variety of ways people seek spirituality and comfort. He became a proponent of Complementary Integrative Medicine (CIM).

### 2) Patient and family engagement

Capturing the patients’ voices, obtaining patient perspectives and finding out what matters most to patients and families were essential to selecting, planning and implementing PCC initiatives. This was achieved in multiple ways. First, facilities had formal mechanisms of obtaining feedback, such as open meetings for patients to speak with hospital leadership, patient surveys, and inviting patients to serve on PCC committees. For example, one site used surveys to ask patients what they would like to see done with funds received to improve the facility. This site also reported involving patients in hiring decisions for key positions.

Second, informal communication between staff and patients was viewed as an invaluable mechanism to receive feedback. While unstructured in most facilities, this was viewed to be a critical first step towards PCC.

*“[Asking for feedback] helps create a relationship between the patient, the family, and their caregivers.” (SM).*

This relationship was seen as a critical aspect of providing PCC, and through ongoing informal interactions with patients and their families, providers learned what they deemed most important for their care.

Finally, participants described different ways of communicating and marketing information to patients about novel PCC innovations. At some COIs, informational signs detailing specific PCC activities were placed around the facility, thereby orienting patients to the Veteran-centered nature of the facility. For example, a sign at one facility stated:

*“This healing environment was created for Veterans to experience activities that embody the spirit of personalized, proactive, patient-driven care.”*

Such signs orienting both staff and patients to the innovations driving PCC cultural change were present throughout the facility. Staff also noted the importance of directly communicating about initiatives with patients. Yet staff and leadership reported challenges finding time to do so in typical clinical encounters.

### 3) Staff engagement

Getting staff engaged with PCC innovations involved a process of enculturation to change attitudes and priorities. It was critical that staff see PCC as essential to care, not as another fleeting VA initiative. This relied on explicit PCC trainings and frequent messaging. One leader noted: critical.

*“We didn’t want employees, patients, and stakeholders to think, ‘Well, that was just a flavor of the month’….that’s just something we did for a year or so, and now we’re going to move on to something else.” (SM).*

Another participant emphasized the need to meet with staff in person to facilitate engagement.

“*Go in person as much as possible. Walk down the hall and share the vision of what you’re trying to do and ask what they think…Build consensus from the beginning.” (MM).*

This demonstrated the commitment of leadership, but more importantly provided an opportunity for staff to provide input and get them engaged in the process.

Staff training was one mechanism for reinforcing cultural change. One attending physician described how he incorporated PCC into his training of residents to make it clear that PCC was inherent to all clinical tasks. Facilities conducted PCC specific trainings to encourage staff to integrate PCC practices into their jobs. These trainings emphasized that PCC was an extension of current practices and attitudes, rather than wholesale replacement of current ‘wrong’ practices. Moreover, one site had begun developing higher level trainings and staff meetings to reinforce the messages and ensure continued engagement in providing PCC. One key strategy in training staff was to capitalize on staff’s emotional connections through the use of Veterans’ stories of illness and VA healthcare. For example, one site created a video featuring a homeless Veteran describing how his life had improved since being in VA care. As one participant explained,

*“What he said on that video just always brings tears, because of the caring and partnering with him to identify what his priorities are.” (SM).*

Thus, the video engaged staff emotionally in the mission of taking care of Veterans. Another site discussed having Veterans tell their stories at orientation and training sessions.

Emotional connections were also built at staff trainings by asking participants to describe their own, or family members’, healthcare experiences. As one participant explained,

*“I think what’s the turning point for people is a personal experience. When we go to our retreats, that’s the first thing in our retreat….We make people talk about an experience, and all of a sudden the light bulb goes off.” (FLP).*

Another strategy for enculturating staff was to infuse PCC through multiple media modalities. Sites messaged staff about PCC frequently using computer screen savers, regular e-mails and bulletin boards. Participants at several sites described awards given to staff when their practices reflected PCC. For example, at one site the “patient-centered employee of the month” award was distributed. One PCC leader stated that she could see the effect of the work: recent award nominations referenced more of the criteria for PCC that leadership had been encouraging.

Finally, opportunities to reflect on patient care practices fostered PCC. Several sites conducted “literature and medicine seminars” – in-depth discussions of literature pertaining to patients’ experiences. A participant noted,

*“We talked about ‘The Diving Bell and the Butterfly’, what it’s like to be a patient who can’t communicate. And that had a profound effect on me. The series is remarkable.” (FLP).*

The same participant also commented favorably about Schwartz rounds [18], a multidisciplinary forum where clinical caregivers discuss social and emotional issues that arise in caring for patients.

*“The cases are usually very challenging, and … and you don’t know how you would have done it differently, because the patients are so difficult and there’s no answer. But to listen to it, they’re very well done, and there are a number of people on the panels.” (FLP).*

### 4) Focus on PCC innovations

Novel patient-centered innovations of varying magnitude and scope were initiated by all levels of personnel: senior management, middle management and front-line staff. Notably, there were two ways in which success required involvement by all.

First, innovations that started with senior leadership required mid-level champions and staff involvement to be successful.

*“[The medical director] ‘paints the landscape,’ and says: ‘Make it happen.’ Then service chiefs pick people to engage in PCC activities and that’s how front line staff become involved. [New initiatives] are occasionally mandated by leadership, and when they are, they are likely to get eye-rolls. It’s easier to have a physician champion introduce them, show that they work, be enthusiastic and willing to put in the effort: this translates to the staff.” (FLP).*

This could be seen at one primary care clinic that was implementing a novel personal health planning tool. Staff interviewed agreed that while the innovation started as a leadership initiative, they were fully involved in designing the process for implementation. The clinic leader collaborated with front line staff to revise the tool to improve ease of use and strategize to integrate its use into clinic flow. When asked who started the personal health planning pilot, one MM underscored this shared ownership of the initiative, saying “*Everyone you talk to will say that they did it.”*

Second, it was important to start with simple projects that would provide early wins. These types of innovations allowed for an early introduction to patient-centered care and could be implemented quickly, and when successful could further garner the engagement of staff. When leadership was receptive and willing to take a chance in supporting a novel idea, they found “quick wins” and created “sparks” of innovation throughout the hospital. In this way, PCC was thought to be ignited in one area of the hospital and then spread to others. One participant noted:

*“You have to pick a project, like noise or customer service. It’s one small project and I think what I learned from our director…was the change is going to evolve over years.” (MM).*

One site fostered these early wins through a unit designation program. Individual units could apply for designation by indicating what steps they were taking locally to improve PCC. Specific innovations came from the units themselves, and were not dictated by the leadership. Designated units received public accolades and a sum of money to make specific improvements on their units.

### 5) Alignment of staff roles and priorities

Staff described challenges they faced incorporating PCC practices and innovations into daily healthcare practices. When PCC was associated with implementing specific innovations, participants struggled with increased workload without alleviation of existing work burdens. Staff perceived a constant stream of shifting prioritized initiatives, and in conjunction with other clinical responsibilities. One MM reflected, *“We are all fractured like an exploding star.”* Participants reported that some staff viewed practicing PCC as an additional, collateral duty which was not well aligned with their top priorities.

*“Some nurses do not feel that it’s aligned, because they’re saying, ‘You’re talking about… aromatherapy… and I’m trying to get a new bed so that my patient won’t fall.’” (MM).*

Clinical performance measures were another competing priority. The time needed to address traditional clinical performance measures on which they were evaluated interfered with attending to PCC. As one participant explained,

“*How can I really be patient-centered, and how can I really sit there and listen deeply when I’ve got about 11 s to do it?” (FLP).*

Providers not being held equally accountable for providing PCC made it appear less valued by the organization. Leaders did recognize this potential conflict. In response, they spent time trying to emphasize that PCC didn’t require a big shift in priorities, and viewed the changes to be integral to providing care.

### 6) Organizational structures and processes

Organizational structures and processes mandated by VA and individual medical centers were often barriers to implementing PCC innovations and this was noted especially by senior and middle managers. Some participants noted that VA rules and regulations sometimes seemed at odds with a patient-driven approach to care.

“*Being bound by rules can steer the decision making process, instead of the very human desire to help people.*”*(SM).*

Providers remaining in organizational silos impaired abilities to provide patient-driven care. Finding *‘flexible’* and creative ways to capitalize on synergies among multiple VA services and initiatives was cited as a key strategy.

*“The messy work to integrate care instead of keeping up the silos that separated services [is] the only way to do PCC.” (SM).*

The hiring processes in VA also made it difficult to hire PCC staff. One participant describing the issues with staffing noted that, *“It may not be possible to weed out the bad seeds,”* referring to employees who did not exemplify PCC attitudes. Hiring complementary integrative medicine staff such as massage therapists or acupuncturists was hindered by issues with determining position specifications and which service would be responsible for such staff. One facility solved this by identifying existing staff with some of those capabilities.

### 7) Environment of care

Changes in the environment of care represented a non-trivial investment and mobilization of resources. One facility, for example, focused efforts on changing the environment of care for women Veterans, creating a separate mammography suite to help them feel more comfortable coming to the VA for women’s health needs. Some participants argued that environmental changes alone might not actually have an impact on patient-centeredness with one noting that: “patient-centered care is more than just the smell of cookies.”

Yet many viewed such changes as important for both staff and patient experiences. As one participant stated:

*“How a patient feels about a place upon entering dictates how he/she feels about the entire experience.” (MM).*

Environment of care changes were seen as most effective when accompanied by functional changes that facilitated patient-provider interaction. Single patient rooms were said to improve communication with providers and facilitate healing. One primary care clinic re-designed spaces to minimize unnecessary patient movement through the clinic and have mental health, social work and complementary medicine providers in close proximity. This was thought to facilitate team collaboration and encourage patient engagement with these providers.

## Discussion

Patient-centered care cultural transformation is a complex and long-term endeavor. Our study revealed that efforts to transform the culture of care must be multifaceted and occur at multiple levels of the organization, including leadership. Efforts should work toward enculturating staff, encouraging innovation, addressing staff priorities, addressing policies and procedures that may interfere with PCC and incorporating the patient perspective in innovations. Our findings provide a deepening of understanding of how culture might change, the role of leadership in this change and other key domains that must be addressed to ultimately impact the ways in which healthcare professionals care for patients. Further, while PCC is often addressed solely at the level of engaging patients in their health care at the clinical level, our study demonstrates that change at all levels of the organization are required for a patient-centered approach.

Our findings regarding the importance of leadership, and engaging staff, patients and families mirror findings from other studies of organizations leading in PCC [11, 19]. Having a committed leadership was frequently discussed as being important for success in all other domains, such as fostering innovation and engaging staff. Our findings enhance the leadership literature by demonstrating the importance of having leaders model patient-centered care, participate in frequent direct communication with staff and encourage risk taking to foster acceptance of PCC. We saw that leadership support of feasible, easily integrated innovations could provide a foothold for subsequent innovation. But not all leaders may be on board with PCC as a high-priority initiative. For some facilities, it was a change in leadership with a new director who had already believed in PCC. For those facilities where leadership is well established, it may take some time learning about the success of PCC initiatives, such as early wins, in order to garner buy in. Having leaders experience high performing PCC facilities may help; when it’s not possible for leaders to experience PCC directly, sharing stories of PCC impact and success in implementation may be keys to garnering leadership support.

These domains may work in distinctly different time frames. Enculturating staff and getting them engaged and on-board with PCC transformation takes time. Notably, sites that were further along were engaging staff repeatedly, with development of higher level trainings and the use of frequent messaging. Similarly, leadership support could take time to build up, and as we note, doing so may take repeated exposure to PCC practices. In contrast, small initiatives in discreet contexts in the medical center may be the earliest wins, and lead to the ‘sparks’ of PCC transformation.

Other studies have identified the importance of measurement and feedback regarding patient experiences. The sites we evaluated were only engaged in more informal mechanisms, such as town meetings, to obtain feedback from patients. We also found that the lack of accountability and incentives was a barrier to engaging staff in PCC practices. Organizations that have such incentives in place may be more effective in transforming care [10, 11].

In our evaluation, we found that facilities were able to engage leadership and staff. Nonetheless, PCC alignment and integration within the organization, particularly surrounding roles, priorities and bureaucratic rules, remained major challenges. Notably, the VA used our findings to create policy level incentives to change by incorporating theses seven domains into senior executive performance measures [20]. Basing metrics for changing organizational processes on studies such as ours may yield greater success in fostering change.

One systematic review of large-system change argues for engaging individuals at all levels of the organization to lead the change, establish feedback loops, attend to local history, engage physicians, and involve patients and families [7]. Extensive research on transforming primary care into patient-centered medical homes found that a focus on shifting roles and transforming mental models through staff trainings were essential [8, 9, 21]. Whereas much of the current work focuses on ambulatory care, patient-centered medical homes, our study begins to examine how PCC can spread beyond primary care to an entire healthcare organization. Several have focused on policy and legislative level mechanisms for fostering change [22]. Our findings demonstrate that change cannot be simply implemented from the top down; rather levers for change in multiple aspects of the organization, from leadership to front line staff, must be engaged for success.

Our study has limitations. The study was conducted in the US Department of Veterans Affairs Medical Centers and these facilities differ from other US medical centers in that it is a government agency, not dependent on insurance reimbursement for services, and also serves a unique patient population. Other healthcare facilities implementing patient-centered care may not encounter as many administrative burdens, but may find identifying resources for implementation to be more challenging.

By studying only medical centers considered leaders in PCC, we may not have uncovered critical barriers that might influence implementation at other facilities. By the very nature of being Centers of Innovation, these centers already had established key leadership support. Site leadership chose to apply; thus we may not have fully captured barriers to implementation from the perspective of those less engaged in PCC. Additionally, although many participants reflected on the history of the transformation, our site visits captured one moment in an evolutionary process. Continuous engagement in the field may yield more insights. Also of note is that some of these sites had been working towards transformation for only 3 years, and this is a first look into the organizational change that was occurring. A longitudinal study of transformation would lead to greater understanding about the how each of these domains contribute to further patient-centered care cultural transformation. Finally, we did not include patient perspectives in this study; future work should include these perspectives to understand how innovations actually impact patient care.

## Conclusions

Although this study examined patient-centered care implementation in VA medical centers, the seven domains we identified suggest a useful starting point for organizations for whom being patient-centered is increasingly a focus of high quality care. Understanding innovation in facilities that are leaders in PCC may be an important start to attaining broader transformation in a large healthcare organization. Even among these leading facilities, some historically supported PCC; others’ efforts were more nascent. Thus we observed variation in the degree to which each facility was engaged in each of the seven domains. Importantly, the findings regarding these seven domains were quickly taken up by VA policy makers and incorporated into key performance metrics for all VA facilities. The use of these domains has thus begun to shape the ways in which facility leaders are working towards implementing PCC. Future work in which measurement of success in attaining PCC would further illuminate those processes that move the needle furthest towards transformation.

PCC requires changing the conversation and interaction between healthcare professionals and patients; however providers work within systems of care that shape these interactions. Epstein et al. argue that achieving PCC depends on three factors: 1) an informed involved patient, 2) receptive and responsive health professionals, and 3) a supportive health care environment [23]. Others are calling for a greater transformation to collaborative care further shifting the roles of patients and providers to more of a partnership [24]. Transforming healthcare systems to focus on these elements and better serve the “whole” patient is a complex endeavor, and as others have argued, requires a systems level approach [25]. Like many other healthcare systems, VA has achieved technically high quality care [26], but our findings indicate more is needed to facilitate a focus on personalized, proactive and patient-driven healthcare.

## Abbreviations

CFIR: Consolidated Framework for Implementation Research
CIM: Complementary Integrative Medicine
COI: Centers of Innovation
FLP: Front-line providers
MM: middle management
OPCC&CT: Office of Patient-Centered Care and Cultural Transformation
OS: Other staff
PCC: Patient-centered care
SM: senior management
VA: Department of Veterans Affairs
